# Exploring Disparities in Atherosclerosis Comorbidity with Aortic Aneurysm

**DOI:** 10.3390/biomedicines13030593

**Published:** 2025-03-01

**Authors:** Aksana N. Kucher, Iuliia A. Koroleva, Maria S. Nazarenko

**Affiliations:** Research Institute of Medical Genetics, Tomsk National Research Medical Center, Russian Academy of Sciences, 634050 Tomsk, Russia

**Keywords:** aortic aneurysm, atherosclerosis, comorbidity

## Abstract

Aortic aneurysm (AA) and atherosclerosis (AS) of various vascular beds are asymptomatic for a long time and are relatively common pathological conditions that lead to life-threatening and disabling complications. In this review, we discuss the current understanding of the high variation in direct and inverse comorbidity of AA and AS as presented in scientific publications. Estimates of AA and AS comorbidity depend on several factors, such as the location of AA (ascending or descending thoracic aorta or abdominal aorta), familial or sporadic cases of AA, syndromic forms of AA, and/or aortic valve pathology (bicuspid aortic valve [BAV]). To identify the causes of the comorbidity of AA and AS, it is important to consider and characterise many factors in detail. These factors include clinical characteristics of the patients included in a study (age, sex) and risk factors (mainly the presence of monogenic forms and BAV, hypertension, hypercholesterolaemia, diabetes mellitus, and cigarette smoking). Additionally, it is essential to consider characteristics of the disease course and the nature of multimorbidity and to take into account pathologies not only of the cardiovascular system but also of other organ systems, with special attention to metabolic and endocrine disorders.

## 1. Introduction

Aortic aneurysm (AA) and vascular atherosclerosis (AS) are diseases that significantly affect global health [1,2,3,4,5]. AA and AS have a number of both common and specific aetiologic factors and stages of pathogenesis. For example, in both AA and AS, various layers of a blood vessel are affected. Nonetheless, in AS, the pathological events are initiated in the endothelium, and then other layers (intima and media) become involved in the process [6,7,8]. On the other hand, in AA at the initial stages, dysfunction of vascular smooth muscle cells (VSMCs) located in the media is observed [9]. Nevertheless, later on, the changes spread to other layers [10].

For these pathologies, a number of common risk factors (e.g., genetics, male sex, older age, cigarette smoking, and hypertension) are known, but specific factors and factors with varying directions of effect have also been found [11,12,13,14,15,16]. The latter include, in particular, type 2 diabetes mellitus (T2DM), which increases the risk of AS but is rare among AA patients. For instance, the risk of abdominal aortic aneurysm (AAA) is two times lower in patients with diabetes mellitus (especially type 2) than in those without T2DM [11,16]. At the same time, some authors consider T2DM a risk factor for AAA but not for thoracic aortic aneurysm (TAA) [17].

Although the monogenic component of both pathologies is known, the spectra of causative genes for them are not identical [14,18,19]. In addition, it is known that for AA, the significance of risk factors (including genetics) differs depending on the affected region of the aorta [14,20]. This pattern is typical not only for TAA and AAA but is also seen in lesions of various segments of the thoracic aorta (aortic arch, ascending and descending aorta) [14]. Finally, in some studies, AS is also classified as a risk factor for AA, but in general, epidemiological data on the comorbidity of AA and AS are ambiguous and sometimes contradictory [21,22,23,24,25,26]. The contradictory opinions concern the importance of AS in AA development, the potential protective effect of AA against AS development, and the causality and/or randomness of the identified relationships between these pathologies.

In this regard, it is important to analyse the link between AA and AS and to identify the causes of contradictory data on the comorbidity of these pathologies. Understanding the pathogenesis of AA and AS and their interrelations is important for optimising preventive measures and for the treatment of patients with isolated and combined forms of these diseases [27,28]. Therefore, the aim of this review is to discuss the data on AS and AA comorbidity and to identify its determinants.

## 2. Sources of Data on the Comorbidity of AA and Vascular AS

For this review, sources of information on the comorbidity of AA and AS included specifically designed studies as well as clinical data presented in publications on AA patients. The publications included in this review cover a substantial period (~15 years), during which approaches to the diagnosis of AA and AS and the clinical description of patients included in a study have changed (Table 1). This state of affairs created some difficulties in comparing the data among research articles. Firstly, a variety of diagnostic techniques have been used to diagnose AS. These have included the presence of AS-associated diseases and data from histological or instrumental examination of the aorta and other arteries. Secondly, the examined patient groups have shown significant variation in the main sampling criteria. In particular, some studies have included patients with AA of specific localisations without taking into account comorbidities and aetiological factors. Furthermore, the studies did not always provide detailed clinical characteristics. In contrast, other studies excluded patients with one [29] or more monogenic forms of TAA [30] and cases with bicuspid aortic valve (BAV) [17]. Furthermore, in some studies, researchers chose more homogeneous groups in terms of aetiologic factors, excluding not only monogenic forms of AA (familial cases) but also AA of an infectious nature, patients with a combination of TAA and AAA, and others [17,31].

It has been suggested that rates of AA and AS comorbidity as well as the significance of risk factors can be influenced by sample heterogeneity and different approaches to diagnosing AS [31]. In addition, the investigated populations exhibited differences in sex and age parameters as well as in other anamnestic data (including the presence of comorbidities, primarily of the cardiovascular system). These differences, on the one hand, could be related to inclusion/exclusion criteria for patients in a study and, on the other hand, could influence the probability of registering the comorbidity of AA with AS. Despite the differences in the approaches used by various authors to design and conduct research, it is currently possible to identify several factors that influence the estimates of AA and AS comorbidity.

## 3. Age of Patients and Comorbidity of AA and Vascular AS

The analysis of AA and AS comorbidity by patient age is of interest because age is considered a significant risk factor for both conditions. Given that these pathologies are age-dependent diseases, the ages of the individuals included in a sample may influence estimates of comorbidity between AA and AS (Table 1).

In a study by L.K. Bickerstaff et al., AS was found in 29% of patients with TAA. The ascending aorta was involved in 51.4% of cases, the aortic arch in 11.1%, and the descending aorta in 37.5%. The age of patients ranged from 47 to 93 years, with a mean age of 65 years for males and 77 years for females [47]. In another paper, patients with sporadic ascending TAA were found to be more frequently diagnosed with aortic AS compared to a control sample, 97% and 73%, respectively [31]. Furthermore, AS featuring a more severe clinical picture [intermediate (types 2 and 3; 35%) or advanced AS (type ≥ 4; 40%)] was more frequently observed in TAA, whereas the majority of control patients showed minimal AS (none or type 1, 80%) [31]. Although the authors of that study applied an age adjustment, it is noteworthy that the control group (represented by organ donors) was younger (56.6 ± 11.4 years) than the sample of TAA patients (62.9 ± 12.0 years).

According to J.S. Seo et al., women who died of external causes had a high severity of AS in one or more aortic segments (95.6%), and aneurysmal changes were not found in any case [48]. At the same time, the age of death was 39 (±14) years there, which is significantly younger than the age of AA manifestation. Additionally, both TAAs and AAAs are less common in the human population [4,49] than AS [48,50]; AA is more common among males [37,51,52], and females are characterised by a later age of AA manifestation [14,47] (the sample in the study by J.S. Seo et al. included only females [48]). In other words, a sample’s characteristics (age and sex of patients) may influence the estimates of AS and AA comorbidity.

The age of patients with AA varied substantially among the studies included in our review, with the mean age ranging from 53 to 75.3 years (Table 1). In addition, in some articles, the age of TAA patients was younger than that of AAA patients. For example, the mean age of AAA patients ranged from 67.8 to 75.3 years, whereas among examined groups of TAA patients, it varied more widely: from 53 to 75 years. In some papers, the mean age of patients was <60 years, namely 53 years [37], 57.6 years [41], 58 years [40], 58.7 years [39], or 59.6 years [29]. On the other hand, some studies on TAA and AAA patients of the same age (73.6 and 73.1 years, respectively) have shown different burdens of AS-associated diseases [17]. In AAA, AS-associated diseases were more frequently used as an estimate of AS prevalence (Table 1). M. Talvitie et al. observed in AAA patients not only an increase in aortic size with age but also more frequent diagnoses of heart disease (not specified) and cerebrovascular events [32]. Higher estimates of the incidence of AS-associated diseases among older AAA patients were reported [36]. Nonetheless, there are also articles showing a different situation: older patients were less likely to have coronary artery disease (CAD) and cerebrovascular diseases [34]. In the work of E.B. Luneva et al. [37], aortic AS was found in 100% of AAA cases.

Among younger TAA patients, atherosclerotic lesions were less common in several studies [38,40]. The highest estimates of the degree of calcification (as a marker of AS) in different parts of the aorta (arch, thoracic aorta, and abdominal aorta) and arteries (left anterior descending coronary artery, left circumflex coronary artery, and right coronary artery) were registered in an older control group (mean age 64.5 years) than in patients with aortic root aneurysm (with annuloaortic ectasia, mean age 59.6 years) and aortic root aneurysm with type A thoracic aortic dissection (63.7 years) [29]. From their results, those authors concluded that aortic root pathology (annuloaortic ectasia or type A dissection) is associated with a decrease in systemic AS. At the same time, there was no clear association between the calcification level of various vessels and age among patients with annuloaortic ectasia and type A aortic dissection (Table 1). Moreover, in another study, measured mean carotid intima–media thickness was significantly greater in men (mean age 69.7 years) with aortic root dilatation compared to healthy control subjects, but no significant correlation was found between carotid plaque and aortic root dimensions [53].

Thus, the age of the patient may indeed influence the estimates of AA and AS comorbidity, but associations between these parameters were not always recorded, indicating the presence of other determinants of such comorbidity risk.

## 4. A Monogenic Component and Aortic Valve Anomalies as Factors in the Formation of Comorbidity Between AA and Vascular AS

The age of AA manifestation is younger in familial cases (in both AAA and TAA) or in the presence of syndromic forms of TAA (Marfan syndrome, Loeys–Dietz syndrome, vascular Ehlers–Danlos syndrome and others), in the presence of pathogenic variants in genes leading to non-syndromic forms of TAA (*ACTA2*, *MYH11*, *PRKG1*, *MYLK*, or others) or in the presence of BAV, which also has an established monogenic basis in some patients (pathogenic variants in gene *NOTCH1*, *TGFBR2*, *MAT2A*, *GATA5*, *SMAD6*, *LOX,* or others) [35,40,43,46,54]. Therefore, it is important to assess how significant the presence of genetic load is for the formation of the comorbidity between AA and AS.

Both AA and AS have a significant genetic load. In approximately 20–30% of patients with TAA, monogenic forms have been identified. In another 20% of cases, there is a familial burden, although pathogenic variants have not been determined [14,55,56]. The familial burden in patients with AAA can exceed 20% [35]. Although no specific causative genes in individuals with this pathology have been described, pathogenic variants characteristic of TAA have been identified in some cases [4]. It has been estimated that familial cases of AAA account for up to 13% of all AAA cases [57]. According to some researchers [58], the total contribution of genetic factors (represented by an additive genetic component) in AAA reaches 77%. Monogenic forms are also known for AS and are mainly represented by hereditary hypercholesterolaemias [59,60], but the set of these genes generally does not overlap with the gene set of monogenic forms of AA. It is important to note that rare missense mutations in the *ACTA2* gene (for example, p.R118Q, p.R149C, and p.R258C/H) are associated with both TAA and early CAD or ischemic stroke [61]. Therefore, the possibility of common genes—and/or regulatory molecules of AA and AS comorbidity—possibly having opposite effects cannot be ruled out.

According to single-cell RNA sequencing (scRNA-seq) data, both AA and AS are characterised by high phenotypic variation among both non-immune cell types (e.g., VSMCs, endothelial cells, and fibroblasts) and immune cell types (e.g., monocytes/macrophages and T and B lymphocytes). The spectrum of aortic cell subtypes depends on several factors, such as the normal or pathological state of health [62,63,64,65,66,67], the stage of the pathological process [68], and the aetiological background (e.g., Marfan syndrome) [69]. The most significant phenotype transformations have been recorded for VSMCs in AA [62,69] and for endothelial cells in AS [64]. Co-clustering (but not completely identical) subtypes of transcriptomically modulated VSMCs have been identified in both pathologies [68]. Furthermore, aortic cells feature not only high plasticity but also transdifferentiation during the development of aortopathies, when either the same cell type can transdifferentiate into different subtypes or functionally similar cells can originate from dissimilar initial cell types [62,70,71,72,73]. Complex interactions between the cells in both AA and AS have been documented too [65,72,74]. These data indicate that it would be worthwhile to perform a more detailed examination of possible cellular and molecular mechanisms underlying the comorbidity between AA and AS, and this topic deserves a separate review article.

Investigated groups of AA patients differ significantly in the prevalence of TAA monogenic forms and of BAV [14,25] (Table 1). In particular, in ~9% of AA cases, a familial origin of BAV has been registered, which features autosomal dominant inheritance with incomplete penetrance, and chromosomal regions (18q, 5q and 13q) have been identified as potential locations of causative genes of BAV [75,76]. BAV is considered a risk factor for TAA. In patients with BAV, noninflammatory degenerative changes (elastic fibre fragmentation, VSMC death, and/or mucoid extracellular matrix accumulation) are observed in the aortic wall. These alterations lead to progressive aneurysmal dilation of the ascending aorta, valve incompetence, and wall dissection [76].

In familial cases of AA, the presence of monogenic forms of TAA and/or cases of BAV has been associated with a younger age of patients and a lower atherosclerotic burden.

When monogenic forms of TAA and/or cases of BAV are present in familial cases of AA, not only a younger age of patients but also a lower atherosclerotic burden have been registered in such patient groups (Table 1). The degree of carotid AS has been found to be less severe in familial AAA cases when compared to sporadic ones (intima–media thickness of 0.89 and 1.00 mm at ages 67.8 and 70.2 years, respectively) [35]. In a sporadic form of ascending TAA, some authors identified aortic AS in 97% of patients (mean age 62.9 years) [31], while another study revealed coronary AS in 34% of patients (at a similar age, 63.0 years) [30]. In the former paper [31], a histological examination of the aorta was performed, and patients with known monogenic forms of AA and BAV were excluded; in the latter article [30], clinical manifestations of coronary AS were evaluated, and only monogenic forms of AA were excluded (information on the presence of BAV was not provided).

It is known that, firstly, an aortic valve pathology is often detected in TAA patients, particularly in the ascending section [31,37,38,40,41]. Secondly, its presence is associated with a lower prevalence of AS than in the case of a tricuspid aortic valve (TAV) (Table 1).

O.B. Dolmaci et al. noted significant differences between patients with BAV (mean age 58.2 years) and those with TAV (64.8 years) in aortic diameter (more than 45 mm: 51.4% and 43.8%, respectively), in the prevalence of AS (62.2% and 87.5%, respectively), and in clinical presentation [25]. In that study, atherosclerotic lesions in the ascending aortic wall were more prevalent and severe in TAV as compared to BAV patients, and this pattern remained significant after correction for the age and sex differences. Furthermore, the calcification score of the coronary artery was higher in TAV patients as compared to BAV patients, although no significant coronary obstruction was observed on angiography [25]. Furthermore, BAV can be registered with high frequency even in control groups and populations [25,43]. Consequently, differences in the incidence of atherosclerotic vascular lesions in groups of both TAA patients and controls may also depend on the ratio of TAV to BAV patients. According to O.B. Dolmaci et al. [25], the lower prevalence of ascending aorta AS in TAA patients, as seen in early studies, may be explained by the high prevalence of BAV (in a study population), which was not taken into account in such projects.

Anatomical features of the aortic valve affect not only the prevalence of AS among patients but also the clinical picture of this disease. In aneurysms with TAV, there has been more frequent and severe AS at the concavity than in the anterior wall of the aorta, whereas in aneurysms with a malformed aortic valve, AS has been less common and did not show any significant differences (from controls) in plaque localisation and severity [23]. C. Doppler et al. have noted that patients with concurrent TAA and TAV have an elevated concentration of lipids in the media, and VSMCs differ in the lipid spectrum from those in patients with concurrent TAA and BAV and from controls (these two groups were more similar to each other in their work) [77]. From this observation, those authors concluded that there are different pathways of TAA formation depending on the type of aortic valve (BAV or TAV) [77], in line with previous opinions [78].

Of note, aortic valve stenosis in patients with TAV has been classified as an atherosclerotic disease; however, on the basis of risk factor analysis and CAD prevalence, TAV with aortic valve regurgitation and BAV with both stenosis and aortic valve regurgitation have not been included in this category [43]. Machine learning models suggest that cardiovascular risk profiles are more predictive of AA in TAV patients than in patients with BAV, and these types of aortic valves imply different pathways to aneurysm formation [79]. Nonetheless, K.D. Boudoulas et al. [80] have reported a high incidence of coronary AS in patients with aortic stenosis in both TAV and BAV, and that coronary AS with BAV (but not in TAV) requires coronary artery bypass grafting more often than in the general population.

BAV is characterised by a significant genetic component, and monogenic forms have been identified in multiple studies [14,75,76]. Additionally, an analysis of gene expression in individual cells of aortic tissue from patients with BAV has revealed several potentially pathogenetically significant molecular markers of BAV development [66]. It is possible that it is the specificity of monogenic and polygenic backgrounds in patients with TAV and patients with BAV that determines the nature of AS and AA comorbidity. The inconsistency of estimates of AS detection in BAV may be attributed to the complex aetiology of this aortic malformation.

Despite the relative rarity of monogenic forms of AA, available data suggest that the comorbidity of TAA with AS can depend on the presence of pathogenic variants in genes of TAA-associated syndromes (Table 1). For example, in patients with ascending TAA in the absence of atherosclerotic changes in the aorta, both monogenic syndromes (3.9%) and BAV cases (37.2%) have been registered the most often, whereas monogenic syndromes have not been detected in patients with AS predominance in the aorta, and the prevalence of BAV has been two times lower [40] (see Table 1).

In a study by J.S. Vapnik et al. [38], monogenic TAA forms and BAV were registered only in patients with ascending TAA (but not with descending and mixed types), and these patient groups had a lower mean age of patients who had AS less frequently (Table 1). For instance, in that work, in patients with ascending TAA, atheroma was observed in 9% of the cases, and calcium deposition in the aortic wall in 8% of the cases. By contrast, among patients with descending TAA, these parameters were 88% and 80%, respectively. These two groups of patients differed in clinical features: in isolated descending TAA (just as in the case of mixed aneurysms involving the ascending and descending sections), patients were more likely to have aortic events (aortic dissection/rupture) and higher mortality [38].

The presence of common genetic causes (of AA and AS) having different directions of effects on AA as compared to AS may be associated with a decrease in systemic AS with aortic root dilation [29]. Moreover, there are recently published studies [26,81] based on data about carotid intima–media thickness, the prevalence of CAD and myocardial infarction in patients with ascending TAA, and the inverse correlation between low-density lipoprotein cholesterol levels and the presence of ascending TAA. The authors of those studies have concluded that TAA acts as “lifelong protection from AS”, and the mutations that cause the aneurysm, while pro-aneurysmal, are also anti-atherogenic [26]. Possible molecular pathways of pro-aneurysmal and anti-atherogenic effects of ascending TAA, which include impaired VSMC phenotypic switching and associated pathways of matrix metalloproteinases (MMPs) and transforming growth factor-β (TGF-β), have also been suggested [81]. Thus, the prevalence of AS among patients with TAA may be influenced by the prevalence (in examined groups) of patients with different locations of AA and, accordingly, the proportion of individuals with Mendelian forms of TAA and BAV.

The presence of pathogenic variants in genes of Mendelian forms of TAA affects not only the risk of AS but also the histological and clinical features of the aorta. In such patients, differences in the spectrum and significance of cardiovascular disease risk factors have been found (Table 1). O. Leone et al. detected atherosclerotic changes in 22.3% of patients with TAA, and these alterations had a complex histological picture, wherein AS was concurrent with degenerative changes of the aorta in 15.2% of the patients, with aortitis in 1.1%, and with both of these pathologies in 2.3% of the patients [40]. Moreover, in that work, Turner, Marfan, and Lois–Dietz syndromes were diagnosed only in patients with the standalone form of TAA, and BAV was also often detected in this group (37.2%), whereas patients with TAA concurrent with AS were more likely to present with hypertension, hypercholesterolaemia, diabetes mellitus, CAD, and current smoking. Additionally, the prevalence of diverse histopathological profiles of TAA differed among subgroups of patients depending on the presence of AAA: in AAA, degenerative disorders accounted for 33% of the cases, mainly atherosclerotic disorders accounted for 37%, and aortitis with/without AS constituted 30%, whereas these histopathological profiles in patients without AAA were registered in 71%, 17%, and 12% of the cases, respectively [40].

The structure of cardiovascular risk factors and comorbidity with cardiovascular diseases varies among groups of TAA patients depending on the proportion of individuals with monogenic forms and/or BAV included in a study. In the presence of such pathologies, patients with TAA tend to have less frequent traditional cardiovascular risk factors such as hypertension, T2DM, and hypercholesterolaemia (Table 1). In particular, compared with individuals with TAV, patients with BAV are less likely to receive a diagnosis of arterial hypertension (87% and 23%, respectively) and of AS (56% and 26%, respectively), but more frequently receive a diagnosis of aortic dilatation (17% and 49%, respectively) and severer valve stenosis (aortic valve replacement in 6% and 23% of the patients, respectively) [46].

In the presence of AS in patients with TAA, the structure of factors is closer to that for diseases associated with AS (Table 1). In some papers, even though TAA was not associated with an increased coronary atherosclerotic burden, hypertension was more frequently found in these patients compared to the general population (61.4% vs. 32.2%, *p* < 0.001), but the prevalence of diabetes mellitus was lower (1.4% vs. 13.1%, *p* = 0.001) [43].

Furthermore, it has been suggested that the presence of atherosclerotic lesions may serve to protect against the development of a serious complication, namely aortic dissection [28,42]. For example, in most patients with thoracic aortic dissection, no progressive AS has been observed, despite the presence of non-progressive intimal lesions (and these findings have not been dependent on sex, age, or a history of hypertension) [42]. V. Stejskal et al. [28] have demonstrated that atherosclerotic changes in the ascending aorta are more prevalent and severe in the group of patients with aneurysm and TAV, compared to patients with aortic dissection (both with TAV and deformed aortic valves) and compared to patients with AA in the presence of valve defects. Thus, the estimates of AS and AA comorbidity depend not only on age but also on the prevalence of monogenic forms of AA and cases with BAV within the examined groups.

## 5. Location-Related Features of the Comorbidity of AA with Vascular AS

The location of the aneurysm (aortic root, ascending, descending thoracic aorta, or abdominal aorta) is an important parameter affecting AA and AS comorbidity (Table 1). First of all, differences in the comorbidity of AA and AS have been observed in relation to the thoracic and abdominal aorta.

AS is considered an additional risk factor for AAA but not TAA [14]. AS is detectable in 25–50% of patients with AAA [12]. A correlation between the development of an atherosclerotic plaque, aortic wall thinning, and abdominal aorta dilation has been established [82]. At the same time, the authors of the cited paper observed individual differences in the response to plaque formation in the abdominal aorta, depending on the nature of the development of the pathological process. If plaque deposition associated with local enlargement leads to thinning of the media and loss of medial elastic lamellae, then conditions that promote aneurysm formation are created. Without artery wall dilation, plaque deposits may predispose the aorta—in the event of continuing plaque accumulation—to stenosis [82]. It has been suggested that AAA is not so much a consequence of AS but rather a focal representation of a systemic disease of the vasculature [83]; some patients who have been referred for examination because of a peripheral arterial disease, transient ischaemic attack, stroke, or internal carotid artery stenosis (especially those with advanced age and high risk) have been advised to get screened for AAA [84].

Nevertheless, there are also opposing opinions regarding the relationship between AAA and AS. For instance, results of a meta-analysis of all available publications (at the time of the study) indicate a negative association of peripheral arterial diseases (for which AS is the most common cause) with AAA growth [85]. A recent study showed that in the presence of athero-occlusive diseases (AODs: CAD, stroke, peripheral arterial disease, or ankle brachial pressure index < 0.90), an increase in AAA volume and diameter is slower than that in patients without such diseases, and the association between AODs and significantly slower AAA growth persisted after adjustment for risk factors and medications [24]. Thus, although AS is often regarded as a risk factor for AAA, there are still unresolved issues regarding the prevalence, causality, incidence, or clinical significance of AAA and AS comorbidity. AAA is also characterised by a familial burden, as in the case of TAA; in patients with a family history of and a genetically determined aneurysm, the risk of comorbidity with AS is lower. Therefore, the inconsistent conclusions about AAA and AS comorbidity may also be related to different proportions of patients with genetically determined AAA cases in the examined groups. Nonetheless, the comorbidity of AAA and AS has not been evaluated in relation to the familial burden.

Two aetiologically distinct subgroups have been identified in TAA that differ in the location of the pathological process. Aneurysms of the aortic root and ascending aorta are often inherited and/or congenital, and the development of aneurysms of the descending aorta has been associated with AS (often referred to as “degenerative”, not related to connective tissue disorders) [14,86]. For example, it has been reported that the size of atherosclerotic plaques in the descending aorta correlates with a greater descending aorta diameter (a 0.18 mm increase in diameter per 1 mm increase in plaque thickness is observed), but non-significant negative associations have been noted between the size of atherosclerotic plaques, risk factors for AS, and dimensions of the proximal thoracic aorta [87]. In the case of ascending TAA, however, especially with a high proportion of patients having monogenic forms of TAA and/or BAV in the studied groups, there has been no connection between TAA and AS [39], or in such patient groups, atherosclerotic lesions have been detectable less frequently [37,38,39,40,41]. Even in the absence of patients with syndromic forms of TAA and BAV in a sample, aneurysms of the root and ascending aorta are diagnosed at a younger age than aneurysms of the descending thoracic aorta: 64.0 and 72.1 years, respectively [38]. There are also differences in the prevalence of atheroma and calcification in AA of the ascending and descending thoracic aorta [38]. In addition, AAA and TAA, just as the ascending and descending sections of the thoracic aorta, are characterised by differences in both the profile and the significance of risk factors [14].

The clinical heterogeneity and interrelations of AA and AS of various locations [12,13,14,88] are connected with a polyaetiological basis and location specificity of molecular, cellular profiles, histological features, and haemodynamic parameters in both intact [89,90,91,92] and affected regions of the aorta [42,93,94,95]. Conditions that either favour or prevent the development of a particular aortopathy may depend on the region of the aorta.

One potential explanation for the divergent effects of both risk factors and clinical pictures of AA and AS may be location specificity of embryonic origin and of function—including those observed at the molecular level—of the aorta’s structural components and of the cells that form them [91,95]. G. Thiene et al. have proposed that the relation between the pathology of the aortic valve (in BAV) and the pathology of the aortic wall (aneurysm development) can be most likely explained by embryological features, because of the commonality of embryonic cells that play a role in the development of both the ascending aorta, aortic arch, and semilunar valves [76]. At the same time, certain similarities (although there are also specific features) of structural and functional features during the ageing of the vascular wall have been found between patients with BAV and those with Marfan syndrome (associated with TAA). These similarities are thinner intima, lower expression of contractile VSMCs, thinner elastic fibres, lack of inflammation, and lower expression of progerin as compared to TAV [96].

Aortic sections show notable structural differences, not only between the thoracic and abdominal aorta but also between individual thoracic aortic segments [97]. These differences may determine the dissimilarities between aortic segments in the phenotype [98] and in the response to damage or degeneration [97,99].

The occurrence of an aortic pathology may be influenced by the microstructural and mechanical properties of an artery, which vary along the arterial tree and are dependent on haemodynamic and geometric features [91]. In particular, the ascending aorta and aortic arch differ in mechanical properties and in the fragmentation index (primarily owing to the complex loading regimes and curved geometry), while micro-structural and mechanical features of the descending aorta show minimal variations [91]. Greater susceptibility to AS in the abdominal aorta is related to a lower number of elastin membranes in the media: their number decreases with the distance from the aortic root (from 50 in the ascending aorta to 20 in the descending aorta) (cited from [86]). The location specificity of various aortic pathologies, including AS and AA, is also associated with the dissimilarity of VSMCs among different segments of arteries [95,100]. Tissues surrounding blood vessels (in particular, peri-vascular adipose tissue) may also influence the risk of both AA and AS [101,102,103]. Thus, the location specificity of properties of aortic sections may act as another determinant of the nature of comorbid relations between AA and AS.

## 6. Conclusions

The reviewed data indicate high variation in estimates of (and conclusions about) AA and AS comorbidity, as presented in scientific publications. The data on the significance of aetiological factors and on the comorbidity of AS with AA (regardless of the location of the pathological process) suggest that AA is a clinically heterogeneous pathology.

Indeed, a number of researchers—on the basis of both their own data and summaries of the results of scientific publications—have come to the conclusion that mechanisms of AA development differ between parts of the aorta [17,25,86]. The differences in mechanisms are confirmed by the fact that analysed groups of AAA and TAA patients possess different atherosclerotic profiles of comorbidities [17].

**Figure 1:**
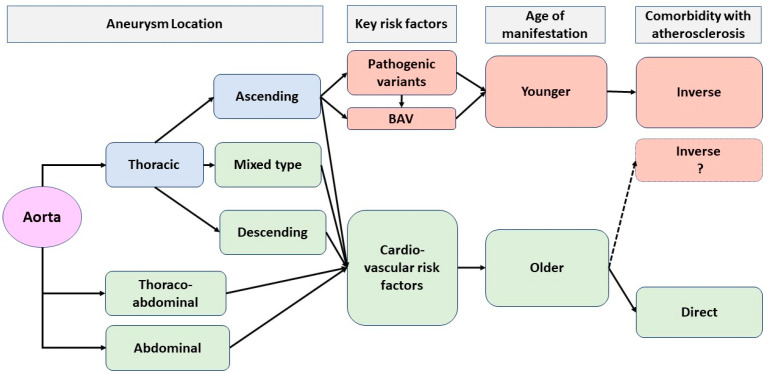
Schematic representation of the importance of different factors for the formation of direct and inverse comorbidity between AA and AS. The dashed line indicates the hypothesised formation of inverse comorbidity between AS and AAA in the case of monogenic and familial forms.

In general, despite the clinical differences between patient groups investigated in different studies (Table 1), it can be said that estimates of AA and AS comorbidity depend on a number of factors (Figure 1): on the localisation of the pathological process (ascending or descending thoracic aorta, abdominal aorta, and the presence of both TAA and AAA in patients), on the proportion (%) of familial or sporadic cases in a sample, and on the presence and frequency of registration of syndromic forms of TAA and/or aortic valve pathology (in particular, BAV). Because the development of any pathologic process is accompanied by molecular and biochemical transformations and depends on which pathologic process arose at an earlier stage (for example, AA or AS), the risk of development of comorbid pathologies and the nature of their course can be determined.

## 7. Future Directions

There are still not enough studies conducted at the level necessary to understand the causes of the formation of direct or inverse comorbidity between AA and AS. From the currently available data on this issue, it can be concluded that further research is necessary, taking into account a number of points. To identify the causes of the formation of AA and AS comorbidity by considering the location of pathological processes and the nature of relations between these pathologies, it is important to consider and characterise a number of factors in detail. These factors include risk factors (primarily the presence of monogenic forms and cases with BAV), clinical features of the patients included in a study, features of the course of this disease, and the nature of multimorbidity. In doing so, it is essential to analyse pathologies not only of the cardiovascular system but also of other organ systems (in particular, metabolic and endocrine). In addition, there is a need for a detailed analysis of structural and functional features and molecular processes occurring during the development of AA and AS in various arteries, and this analysis deserves special attention.

## Figures and Tables

**Table 1:** Comorbidity of AA with AS and other diseases.

Type of Aortopathy (n = Sample Size; % Males/Females)	Age, Years	AS, % {Clinical Features of AS}		Method of AS Diagnostics	Frequency, %							Ref.
					Syndromes	BAV	AH	HC	T2DM	Smoking ^##^	AAA	
AAA 49–54 mm (n = 557; 0/100%)	73.2	Heart disease: 29.4, CVE: 11.1		ND (Medical history, heart disease)	ND	ND	77.9	ND	12.2	74.0	100	[32]
AAA 55–64 mm (n = 1085; 0/100%)	75.3	Heart disease: 32.8, CVE: 12.2			ND	ND	78.3	ND	11.4	69.1	100	
AAA (n = 42; 92.9/7.1%)	63.8	{CAD: 35.7}		ND (Medical history, CAD)	ND	ND	61.9	ND	19	56.1	100	[33]
AAA (n = 108; 90.7/9.3%)	71.7	{CAD: 38.0; HF: 8.3; CVE: 14.8; PAOD: 23.1}		ND (Medical history, CAD)	ND	ND	70.4	77.8	25.0	97.2	100	[34]
AAA (n = 200; 92.5/7.5%)	74.3	{CAD: 34.0; HF: 6.5; CVE: 19.0; PAOD: 15.0}			ND	ND	80.5	83.0	17.5	95.0	100	
AAA (n = 461; 84.6/15.4%)	69.7	{Carotid AS: intima–media thickness: 0.97 mm}		Ultrasonography	Excl.	ND	78.7	94.8	19.5	91.5	100	[35]
Familial AAA (n = 103; 79.6/20.4%)	67.8	{Carotid AS: intima–media thickness: 0.89 mm}		Ultrasonography	Excl.	ND	68.9	91.3	12.6	84.6	100	
Sporadic AAA (n = 358; 86.0/14.0%)	70.2	{Carotid AS: intima–media thickness: 1.00 mm}		Ultrasonography	Excl.	ND	81.5	95.8	21.5	93.6	100	
AAA (n = 108; 83.3/6.7%)	75.1	{CAD: 39.8}		CAD	ND	ND	49.1	ND	17.6	86.1	100	[36]
Healthy controls (n = 12; 83.3/16.7%)	68.8	{CAD: 0}			ND	ND	25.0	ND	0	41.7	0	
AAA (n = 211; 86/14%)	73.1	CAD (53%); 3-vessel coronary disease (41%)		CAD	Excl.	Excl.	81	59	39	88	100	[17]
TAA (n = 132; 74/26%)	73.6	CAD (23%); 3-vessel coronary disease (10%)		CAD	Excl.	Excl.	91	50	23	76	Excl.	
TAA (n = 90; 70/30%)	53	34		{Histology, n = 45: cystic medial necrosis = 55; AS = 27}	9	34	ND	ND	ND	ND	ND	[37]
AAA (n = 30; 90/10%)	69	100		Histology	ND	ND	ND	ND	ND	ND	100	
ATAA (n = 628; 70/30%)	60.3	9 {atheroma: 9; calcifications: 8}; {CABG: 9}		CT and MRI	4	31	59	ND	5	44	1	[38]
DTAA (n = 130; 39/61%)	72.0	88 {CABG: 15}	{atheroma: 88; calcifications: 80}		0	0	82	ND	12	71	67	
MTAA (n = 86; 50/50%)	74.0	ND {CABG: 21}			0	1	80	ND	13	78	51	
TAA (n = 416; 67/33%)	64.0	{CABG: 9}		CT and MRI	Excl.	0	64	ND	6	47	1	
DTAA (n = 130; 39/61%)	72.1	{CABG: 15}			Excl.	0	82	ND	12	71	67	
MTAA (n = 81; 58/42%)	74.3	{CABG: 21}			Excl.	0	80	ND	14	79	52	
Sporadic ATAA (n = 68; 57/43%)	62.9	97 {intermediate AS (types 2 and 3): 35%; advanced AS (types ≥ 4): 40%}		Histology	Excl.	29	65	ND	0	54	ND	[31]
Controls (n = 15; 47/53%)	56.6	73 {minimal AS (none or type 1): 80%}			Excl.	0	67	ND	13	47	ND	
ATAA (n = 111; 66.7/33.3%)	58.7	{rarely associated with AS and with inflammatory infiltration}		Histology	10.8	30.6	39	ND	ND	ND	ND	[39]
TAA (n = 255; 74.1/25.9%)	66	22.1 (Infl.-AS: 3.5; Deg.-AS: 15.2; AS-aortitis: 1.1; Deg.-AS-aortitis: 2.3) {moderate AS: 10.9; severe AS: 11.3; mild AS: 31.7}		Histology	2.5	30.1	82.7	52.5	8.2	46.6	10.5	[40]
ATAA/isolated degeneration (n = 172; 81.9/18.1%)	63	0			3.9	37.2	79.1	52.3	5.8	41.1	5.2	
ATAA/mainly AS (n = 48; 77.1/22.9%)	69	100			0	18.7	87.5	75	16.6	66.6	20.8	
ATAA + aortitis with/without AS (n = 35; 31.4/68.6%)	75	26			0	11.4	94.2	57.1	8.5	45.7	22.8	
ATAA + BAV (n = 77; 75.3/24.7%)	58	{medial degeneration: 83; mainly AS: 12; aortitis with/without AS: 5}			1.3	100	74	37.6	10.3	37.5	0	
ATAA + TAV (n = 178; 73.5/26.5%)	69	{medial degeneration: 61; mainly AS: 22; aortitis with/without AS: 17}			3.2	0	86.5	45.5	7.3	50.5	15.1	
ATAA (n = 163; 69.9/30.1%)	57.6	37.4 (including AS of aorta: 12.9; of coronary arteries: 16.6; of carotid arteries: 5.5; no change: 15%)		Ultrasonography of major arteries, coronary angiography	1.8	44.2	67.5	4.3	5.5	ND	ND	[41]
BAV (n = 37; 78.4/21.6%): 51.4% had TAA	58.2	62.2 {progressive AS: 18.9}		Histology	ND	100	37.8	27.0	0	13.5	ND	[25]
TAV (n = 32; 50/50%): 43.8% had TAA	64.8	87.5 {progressive AS: 50, AS lesions in the ascending aortic wall to be more prevalent and severe in TAV as compared to BAV}			ND	0	53.6	21.9	12.5	21.9	ND	
TAA (n = 33; 81.8/18.2%)	62.7	No difference in prevalence and severity of atherosclerotic lesions between TAA and non-TAA			ND	54.5	54.5	15.2	6.1	21.2	ND	
non-TAA patients (n = 36; 80.8/19.2%)	59.2				ND	65.4	42.3	34.6	15.4	7.7	ND	
BAV (n = 35; 82.9/17.1%): 51.4% had TAA	59.9	{CABG: 11.4}		Coronary angiography, CAGE score for the extent and severity of coronary artery sclerosis	ND	100	42.9	17.1	8.6	14.3	ND	
TAV (n = 24; 79.2/20.8%): 62.5% had TAA	62.9	{CABG: 41.7}			ND	0	58.3	33.3	12.5	16.1	ND	
BAV-TAA (n = 19; ND/ND %)	ND	{CAGE ≥ 20: 0.83; CAGE ≥ 50: 0.44; calcification of the aortic wall: 2.11, calcification of the coronaries: 2.56}			ND	100	ND	ND	ND	ND	ND	
BAV non-TAA (n = 18; ND/ND %)	ND	{CAGE ≥ 20: 1.53; CAGE ≥ 50: 0.35; calcification of the aortic wall: 2.35, calcification of the coronaries: 3.47}			ND	100	ND	ND	ND	ND	ND	
TAV-TAA (n = 14; ND/ND %)	ND	{CAGE ≥ 20: 1.8; CAGE ≥ 50: 1.53; calcification of the aortic wall: 2.37, calcification of the coronaries: 7.67}			ND	0	ND	ND	ND	ND	ND	
TAV non-TAA (n = 18; ND/ND %)	ND	{CAGE ≥ 20: 2.72; CAGE ≥ 50: 1.83; calcification of the aortic wall: 3.56, calcification of the coronaries: 8}			ND	0	ND	ND	ND	ND	ND	
Aortic root aneurysm: annuloaortic ectasia (n = 31; 61.3/38.7%)	59.6	{Calcification: LAD: 58; LCA: 26; RCA: 19; arch of aorta: 45; thoracic aorta: 48; abdominal aorta: 65}		Calcification in coronary arteries and aorta was used as a marker of AS and was quantified by evaluating CT scans	Excl.	ND	87	45	10	48	ND	[29]
Aortic root aneurysm: type A ascending aortic dissection (n = 33; 54.5/45.5%)	63.7	{Calcification: LAD: 42; LCA: 21; RCA: 12; arch of aorta: 58; thoracic aorta: 58; abdominal aorta: 57}			Excl.	ND	85	48	6	70	ND	
Controls (n = 86; 53.5/46.5%)	64.5	{Calcification: LAD: 59; LCA: 37; RCA: 28; arch of aorta: 64; thoracic aorta: 70; abdominal aorta: 71}			Excl.	ND	59	24	21	41	ND	
Sporadic TAA (n = 144: ATAA, n = 72; aortic bulb, n = 16; ATAA and aortic bulb, n = 56; 74.3/25.7%)	63.0	AS coronary syndrome: 34.0		Medical histories (AS coronary syndrome)	Excl.	ND	79.1	22.9	15.3	45.1	ND	[30]
Controls (n = 90; 62.2/37.8%)	61.1	0			0	ND	31.1	15.6	13.3	51.1	ND	
AAD, Type A (n = 51; 51/49%)	62.5	13.7: normal intima, 72.6: non-progressive intimal lesions, 13.7: progressive intimal lesions		Histology	ND	0	59	ND	0	ND	ND	[42]
Controls (n = 17; 47/53%)	63	0: normal intima, 17.6: non-progressive intimal lesions, 82.4: progressive intimal lesions			0	0	ND	ND	ND	ND	ND	
TAA (n = 70; 74.3/25.7%)	64	Coronary AS: CAGE ≥ 20: 1.65; CAGE ≥ 50: 0.98		Coronary angiography, CAGE score for the extent and severity of coronary artery sclerosis	ND	35.7	61.4	21.4	1.4	48.5	ND	[43]
Non-TAA (n = 169; 72.8/27.2%)	62	{CABG– 15.7}			ND	36.7	64.5	26	9.5	45.5	ND	
General population	ND	Coronary AS: CAGE ≥ 20: 2.03, CAGE ≥ 50: 1.42		ND	ND	ND	32.2	18.3	13.9	ND	ND	
BAV (n = 87; 82.2/17.8%)	54	{CABG: 20.7}			ND	100	57.5	20.7	2.3	ND	ND	
General population	≈54	ND		CAD	ND	ND	16	9.1	6.6	ND	ND	
TAV (n = 152; 67.8/32.2%)	67	CAGE ≥ 20 (not CAGE ≥ 50) is lower than in TAV ($)			ND	0	67.1	27	9.9	ND	ND	
General population	≈67	{CAD: 3}		CAD	ND	ND	40.9	22.7	16.9	ND	ND	
TAA (n = 135; 63.7/26.3%)	72.1	mild or moderate AS of the aortic arch: 71.85; severe AS of the aortic arch: 28.15		CT scan and echocardiography	ND	ND	ND	ND	ND	ND	ND	[44]
Controls (n = 5; 60/40%)	66	grades I-II of AS: 20		Histology	Excl.	Excl.	40	40	60	ND	ND	[45]
ATAA, MAD = 4.5 cm (n = 5; 80/20%)	66.2	grade 0 of AS: 100			Excl.	Excl.	60	60	40	ND	ND	
TAV-TAA, MAD ≤ 6 cm (n = 4; 50/50%)	65	100 {grades I-II of AS: 50; grade II of AS: 50}			Excl.	Excl.	75	50	0	ND	ND	
TAV-TAA, MAD > 6 cm (n = 6; 50/50%)	75.3	100 {grade I of AS: 50; grades III–IV of AS: 16.7; grades IV–V of AS: 33.3}			Excl.	Excl.	83.3	16.7	0	ND	ND	
MAS, MAD = 6.9 cm (n = 5; 20/80%)	68.6	100 {grade IV of AS: 40; grade V of AS: 20; grade VI of AS: 40}			Excl.	Excl.	100	40	0	ND	ND	
BAV + type A aortic dissection (n = 47; 77/33%)	58	24 {mild: 17; moderate: 7; severe: 0; CABG: 26}		Histology	6	100	68	49	9	66	ND	[46]
TAV + type A aortic dissection (n = 53; 76/24%)	66	51{mild: 43, moderate: 6; severe: 2; CABG: 30}			0	0	87	49	11	60	ND	

Note: $ After multivariate analysis with adjustment for the body mass index, diabetes mellitus, hypercholesterolaemia, previous myocardial infarction, unstable angina pectoris, and a family history of CAD and of aortic dilatation; ^##^—former or current smokers; AAA, abdominal aortic aneurysm; AAD, ascending aortic dissection; AH, arterial hypertension; AS, atherosclerosis; ATAAs, isolated ascending thoracic aortic aneurysms; BAV, bicuspid aortic valve; CABG, coronary artery bypass grafting; CAGE scores the severity and distribution of coronary obstruction and denotes non-obstructive disease (CAGE  ≥  20, coronary obstruction of 20–49%) or obstructive disease (CAGE ≥ 50, coronary obstruction of  ≥  50%); CVE, cerebrovascular events; Deg., degenerative; T2DM, diabetes mellitus; DTAAs, isolated descending thoracic aortic aneurysms; Excl., patients with a diagnosis of a known genetic aortic aneurysm syndrome were excluded; HC, hypercholesterolaemia; HF, heart failure; Infl., inflammatory; LAD, left anterior descending coronary artery; LCA, left circumflex coronary artery; MAD, mean aortic diameter; MAS, mega-aortic syndrome; MTAA, mixed thoracic aortic aneurysm (combined AAA and DTA); ND, no data; PAOD, peripheral arterial occlusive disease; RCA, right coronary artery; TAA, thoracic aortic aneurysm; TAV, tricuspid aortic valve.

## Data Availability

No new data were created or analysed in this study. Data sharing is not applicable to this article.
